# Quality of antenatal and delivery care and postnatal care use: A multi-country observational study of 400,000 births

**DOI:** 10.1371/journal.pmed.1005055

**Published:** 2026-04-21

**Authors:** Jan E. Cooper, Hwa-Young Lee, Katherine Wright, Prashant Jaryan, Margaret E. Kruk

**Affiliations:** 1 Department of Epidemiology, Biostatistics and Occupational Health, Faculty of Medicine and Health Sciences, McGill University, Montréal, Canada; 2 Department of Global Health and Population, Harvard T.H. Chan School of Public Health, Boston, Massachusetts, United States of America; 3 Graduate School of Public Health and Healthcare Management, The Catholic University of Korea, Seoul, Republic of Korea; 4 Catholic Institute for Public Health and Healthcare Management, The Catholic University of Korea, Seoul, Republic of Korea; 5 Centre for Chronic Disease Control, New Delhi, India; 6 Department of Medicine, Division of General Medicine and Geriatrics, Washington University School of Medicine, St. Louis, Missouri, United States of America; Burnet Institute, AUSTRALIA

## Abstract

**Background:**

Postnatal care (PNC) plays a crucial role in averting newborn mortality, yet its use remains low, particularly in regions with the highest mortality. While demographic and social determinants of PNC use have been well studied and have informed current strategies focused on changing care-seeking behaviors, the stagnating decline in neonatal mortality highlights the need for upstream and system-wide approaches to increase PNC uptake. Limited evidence exists to guide system-level reforms; therefore, we investigated whether health systems that ensure high-quality perinatal care are associated with increased use of PNC.

**Methods and findings:**

We performed a cross-sectional observational study using Demographic and Health Survey data from 38 countries that had not met SDG neonatal mortality targets by 2020. The study population comprised women aged 15–49 years whose most recent live birth occurred within five years preceding the survey. We employed logistic regression models with country fixed effects to examine associations between: (1) perinatal service utilization, (2) service quality; and their relationship with postnatal checkups for newborns within 28 days. We analyzed utilization-quality interactions to determine how the effects of service coverage on PNC use varied by care quality and conducted wealth-stratified analyses to assess how quality effects on PNC use differed across socioeconomic groups. High-quality perinatal care was associated with a 2-fold increase in the probability of postnatal checkups within 28 days (0.406 in the highest quality tertile versus 0.221 in the lowest quality tertile). For mothers who received the lowest tertile of service quality, full utilization of perinatal services yielded negligible changes in postnatal checkup probability (0.217 to 0.216). Conversely, for mothers who received the highest quality of antenatal and perinatal care, full access to perinatal care improved the probability of postnatal check-ups (0.392 to 0.428). Notably, women in the lowest wealth quintile experienced a substantial increase in postnatal checkup probability from 0.224 to 0.481 between low and high-quality care cohorts, while this differential was less pronounced in the highest wealth quintile (0.236 to 0.450). Key limitations include restricted quality indicators, potential recall bias from self-reported measures, limited information on follow-up care, and the cross-sectional nature of the data, which limits causal interpretation.

**Conclusions:**

Our interaction analysis reveals a critical insight: high-quality care substantially enhanced the magnitude of the association between expanded perinatal service use and PNC use, whereas increased utilization alone was not linked to higher PNC uptake. Notably, the impact of improved care quality was most pronounced among the lowest wealth groups, highlighting its potential as a mechanism for promoting equity. By demonstrating the central role of care quality in promoting PNC utilization, particularly among disadvantaged populations, our findings suggest that improving health system quality could be a more effective strategy for achieving universal maternal healthcare coverage than traditional access-focused approaches.

## Author summary

**Why was this study done?:**

- In many low- and middle-income countries, neonatal mortality remains high and postnatal care (PNC) use, which is crucial to newborn survival, remains persistently low.
- Neonatal mortality and severe intrapartum complications are telling indicators of system quality, as these deaths and complications are preventable with high-quality care.
- Previous research on postnatal care use includes regionally based studies and focuses on access to care and socioeconomic barriers to using care, but limited evidence exists on how health system quality affects care-seeking behaviors for PNC.
- To generate new approaches for improving newborn survival, we need to disentangle the role of the health system, specifically whether high-quality, accessible pregnancy and delivery care affects the use of care for newborns in the first month after birth.

**What did the researchers do and find?:**

- We analyzed data from 432,834 births across 38 low- and middle-income countries that had not met SDG targets for neonatal mortality, examining the relationship between perinatal care quality and postnatal care utilization.
- We analyzed the interaction between perinatal service coverage and quality to determine how the effects of service coverage on PNC use varied by care quality, and conducted wealth-stratified analyses to assess how the quality effects on PNC use differed across socioeconomic groups.
- We found that high-quality perinatal care approximately doubled the probability of postnatal checkups within 28 days (0.406 versus 0.221 for highest versus lowest quality tertiles), while increased service utilization alone showed negligible impact without accompanying quality improvements.
- The benefits of high-quality care were most pronounced among the poorest women, whose probability of postnatal checkups increased from 0.224 to 0.481 between low and high-quality care groups.

**What do these findings mean?:**

- Our findings suggest an expanded role for quality of care: beyond improving newborn outcomes, it also shapes women’s likelihood of returning for postnatal services, demonstrating that quality is essential for access to translate into continued engagement with the health system.
- Improving health system quality throughout the perinatal period could be a more effective strategy for achieving universal maternal healthcare coverage than traditional approaches that prioritize expanding access alone.
- High-quality perinatal care may serve as an equity-promoting intervention, with the greatest benefits observed among disadvantaged populations, potentially narrowing socioeconomic disparities in maternal healthcare utilization.
- The main limitations of this study are that we used limited measures of care quality, relied on mothers’ memories, lacked information on whether follow-up care occurred and on its content, and the cross-sectional nature of the data, which precludes causal inference.

## Introduction

Despite the increase in and widespread use of facility deliveries, neonatal mortality remains higher than national and global targets in many low- and middle-income countries. In 2020, 2.4 million newborns died in the first month of life, accounting for nearly half of all deaths under the age of five [1]. Approximately three-quarters of those deaths occur in the first week of life, typically from preterm birth, intrapartum-related complications, and infections [1]. The bulk of these deaths occur in low- and middle-income countries (LMICs), and are a clear reflection of low access to and poor quality of care enabled by poor quality health systems. Neonatal mortality and severe intrapartum complications are particularly telling indicators of system quality, as these deaths and complications are amenable to high-quality care [2]. Postnatal care (PNC) plays an important role in reducing newborn death within 28 days as it includes preventive assessments to detect and manage complications in mothers and newborns, including umbilical cord care, special attention for high-risk neonates, infection screening, growth monitoring, and educating mothers on recognizing danger signs [3]. However, the use of PNC is variable [3,4].

Patients’ decision to use healthcare is complex, and the uptake of health services is often a combination of user preferences—preferences that reflect their belief in the importance of the care, cultural practices, and intrahousehold decision-making—as well as the cost and convenience of the care [4]. Use of PNC is especially illustrative of the complexities in the decision of using healthcare. Often, caregivers’ time is limited with the care of a newborn, and the decision to spend time and money to receive PNC is predicated on the belief that this care is beneficial. Pregnancy and childbirth are often periods of ongoing contact with the health system for antenatal and later delivery care. As a result, both the quality of pregnancy and delivery care have the potential to shape the perceived value of PNC.

While the antenatal, delivery, and immediate postpartum periods are times of relatively intense use of health services, we know little about whether the quality of care received during this time affects the use of PNC. Furthermore, we lack comprehensive evidence on the health system determinants of PNC use across LMICs where maternal and neonatal mortality are high, and where PNC use remains persistently low. The evidence on the drivers of PNC utilization is based largely on regional studies [5–9]. These studies have predominantly focused on individual-level socioeconomic determinants, such as education level, wealth, and place of residence. Specifically, this evidence indicates that women living in urban areas, those with more education, and who have more wealth are more likely to use PNC. Women’s experience in the health system plays a role too: studies show that the frequency of women’s contact with the healthcare system throughout their pregnancy influences their use of PNC, finding that women who make at least 4 antenatal care (ANC) visits are more likely to use healthcare in the first month after birth [8–10]. A key feature of high- quality maternal and newborn care is continuity that provides preventive care and treatments throughout pregnancy, during delivery, and in the weeks and months after birth. Yet, when the content of care along the maternal, newborn, and child healthcare continuum is poor, the most vulnerable women receive the lowest quality care [11–13].

Taken together, previous work points to the sociodemographic determinants of PNC use, and separately, other evidence highlights the role of quality in predicting future use of health services. Limited research has looked at the combined effects of individual-level factors and the frequency and quality of maternal care on uptake of PNC, in other words, whether effective access to care throughout pregnancy and delivery affects use of care during the postnatal period.

To generate new approaches for improving newborn survival, we need to disentangle the role of the health system, specifically whether high-quality, accessible pregnancy and delivery care affects the use of care for newborns in the first month after birth. Therefore, using data from the Demographic and Health Survey (DHS) conducted in 27 countries that had not met the Sustainable Development Goal (SDG) target for neonatal mortality by 2020, we aimed to investigate whether higher quality care provided during the antenatal and immediate postpartum periods is associated with women’s uptake of PNC in the first 28 days after birth.

## Methods

### Data source, settings, and sample

We used data from the DHS [14] and employed an observational cross-sectional study design. Countries were included in the analysis if they had not achieved the SDG target for neonatal mortality—12 per 1,000 live births—as of 2020 [15] and had data collected in 2015 or later.

DHS is a nationally representative population survey which employs a stratified two-stage sampling design, covering both urban and rural areas. In the first stage, primary sampling units (PSUs)—which are villages or census enumeration blocks—were selected with a probability proportional to their population size. In the second stage, households within each PSU were chosen through systematic random sampling. The study sample was defined as the most recent birth (singleton or multiple) occurring within the five years preceding the survey, based on interviews with women aged 15–49 years.

### Outcome and main interest variables

The main outcome is at least one postnatal checkup for newborns within the first 28 days of life. The key independent variables of interest include measures of adequate utilization and quality of perinatal services during the antenatal period and during birth. Adequate utilization was defined by three main criteria: completing four or more ANC visits during pregnancy, having the first ANC visit within three months of pregnancy, and giving birth at a healthcare institution. Meeting all three criteria was defined as full utilization of perinatal services.

The quality of perinatal services was assessed based on specific service items provided during ANC visits and the immediate postpartum period. Guided by the World Health Organization (WHO) guidelines for ANC and PNC [16,17], we first compiled a list of essential service items to be provided during pregnancy and the immediate postpartum period. These items were then mapped to the corresponding variables available in our DHS dataset. In the DHS, information on these service items was collected through maternal self-reports on the contents of ANC and immediate postpartum care (IPC). A comparison between the service items derived from WHO guidelines and those included in our study is presented in Table A in S1 Appendix.

A total of six essential service items were identified as measures of high-quality ANC: weight measurement, blood pressure measurement, urine sample collection, blood sampling, provision or purchase of iron supplements, and tetanus injection. For high-quality IPC, four items were identified as essential services: newborn weight measurement at birth, examination within 24 hours after birth, examination by a doctor, nurse, or midwife, and initiation of breastfeeding within one hour of birth. ANC and IPC quality scores were calculated as the proportion of service items received out of the total number of items, multiplied by 10 to facilitate the interpretation. A composite quality score of perinatal services was then constructed by combining the ANC and IPC quality scores. The ANC, IPC, and composite quality scores each ranged from 0 to 10.

In the main analyses, the composite score was operationalized as tertiles (low, medium, and high) to enhance interpretability for policymakers and implementers and to facilitate prioritization for policy application, while also accounting for the potential non-linear association between service quality and the probability of a newborn receiving a postnatal checkup. Analyses treating the quality score as a continuous variable were conducted as sensitivity analyses to assess robustness.

### Covariates

Covariates included maternal sociodemographic factors, baby demographic factors, and known medical risk factors for neonatal mortality. Maternal sociodemographic factors encompassed the mother’s age at pregnancy (categorized as <20, 20–35, and >35 years), educational level (no education, primary graduate, secondary graduate, and higher than secondary graduate), empowerment level (scored from 0 to 10), household wealth (categorized into quintiles with the 1st quintile representing the poorest), marital status (currently married and not married), and place of residence (rural and urban). Women’s empowerment was measured with reference to the methodology described in Lewis and colleagues (2022), which includes 25 indicators most likely to be associated with seeking high-quality care for children [18]. Each indicator is binary, with a value of one indicating greater empowerment. A woman’s empowerment score was calculated as the mean proportion of positive responses to these indicators, resulting in a range from zero to one, where a higher score corresponds to greater empowerment. This score was multiplied by 10 to facilitate interpretation.

Baby demographic factors considered in this study included sex (male and female) and birth order (1st, 2nd, 3rd, and 4th or higher). For known medical risk factors of neonatal death, we included the mother’s experience of a previous neonatal death, multiple pregnancies, and delivery by Cesarean section. Additionally, we included neonatal risk factors defined based on small baby size, low birth weight, or preterm birth. Baby size was derived from the question asking “Was your baby very large, larger than average, average, smaller than average, or very small when your baby was born?”, of which “small or very small” were classified as indicating a small baby size. Low birth weight was defined as a weight at birth of 2,500 grams or less. Preterm birth was defined as being born at eight months of gestation or earlier.

### Statistical analysis

We first described the characteristics of the final analytic sample, including the crude rate and its 95% confidence interval (95% CI) of babies receiving postnatal checks within 28 days, segmented by categories of each covariate and by country. Sampling weights were applied to ensure national representativeness within each country. To assess potential bias due to missing values, we compared the characteristics of the final analytic sample with those of the original sample. Because approximately 56% of observations in Bangladesh were excluded due to missing values in neonatal risk factors, comparison between the original and final analytic samples was additionally presented separately for Bangladesh.

We then reported the global and country-specific mean scores for ANC, IPC, and composite perinatal service quality, along with their standard deviations (SD), highlighting countries with scores below the global average. Additionally, 38 countries were ranked by continent according to the full utilization rate of perinatal services. These rankings were visualized graphically alongside the utilization rates of individual components of perinatal services.

The main analyses were conducted using logistic regression models with country fixed effects. First, we examined the association between utilization of perinatal services and the likelihood of a baby receiving a postnatal check within 28 days of birth, including a composite indicator representing full utilization of perinatal services as the key independent variable (Model 1). We then extended this model by incorporating the composite measure of perinatal service quality, categorized into tertiles (low, medium, and high) (Model 2). From model 2, we postestimated the predicted probabilities of a baby receiving postnatal check within 28 days according to full utilization status and quality level.

To assess whether the association between full utilization of perinatal service and receipt of a postnatal check varied according to perinatal service quality, we included interaction terms constructed by multiplying the indicator for full utilization and the categorical composite quality score (Model 3). As sensitivity analyses, we re-estimated the corresponding models by replacing the categorical tertiles with the continuous composite quality score (Model 2-1 and 3-1 in Table F in S1 Appendix).

We further evaluated effect modification by household wealth among mothers who had fully utilized perinatal services. Specifically, we examined the interaction between perinatal service quality and wealth level, modeling quality both as a categorical variable (Model 4 in Table G in S1 Appendix) and, in sensitivity analyses, as a continuous variable (Model 4-1 in Table G in S1 Appendix). Wealth-stratified analyses were subsequently conducted to estimate the association between perinatal service quality (categorized into tertiles) and the likelihood of babies receiving a postnatal check within 28 days within each wealth quintile. Predicted probabilities of a baby receiving postnatal check within 28 days were postestimated from these models and presented graphically.

Finally, we estimated country-specific predicted probabilities of babies receiving postnatal check within 28 days across tertiles of the composite quality score among mothers who had fully utilized perinatal services in each of 38 countries. A complete case analysis was performed, excluding observations with missing data on any analytic variables. Results were presented in both tabular and graphical formats. All analyses were carried out using Stata software, v18.1.

### Ethical approval

The DHSs receive government approval and adhere to ethical standards, including maintaining written record of verbal informed consent and ensuring confidentiality during data collection. This study used publicly available, de-identified data and was deemed exempt from institutional review board review by the Harvard Longwood Campus Institutional Review Board. This study is reported as per the Strengthening the Reporting of Observational Studies in Epidemiology (STROBE) guideline (S1 STROBE Checklist)

## Results

### Analytic sample

A total of 38 countries met the inclusion criteria (the countries and their corresponding survey years are listed in Table B in S1 Appendix). After excluding cases with missing data, the final analytic sample included 432,868 respondents from 38 countries (Table 1). The overall missing rate was 5.3%. A comparison of the characteristics between the original sample (*n* = 475,084) and the final analytical sample revealed minimal differences across the categories of each variable (Table C in S1 Appendix). Notably, Bangladesh had a high missing rate exceeding 50%, primarily due to the unavailability of information regarding baby birth weight, size at birth, and preterm birth status. In Bangladesh, the final analytic sample exhibited a higher socioeconomic status compared to the original sample (Table D in S1 Appendix).

**Table 1:** Characteristics of final analytic sample.

Categories		*N* (%)	% PNC ≤ 28days	(95% CI)
Total		432,868	33.4	(34.3, 33.6)
***Maternal sociodemographic factors***				
Pregnancy age (years)	<20	52,003 (12.0%)	32.4	(31.9, 33.0)
	20–35	338,218 (78.1%)	34.7	(34.5, 35.0)
	≥35	42,647 (9.9%)	29.0	(28.4, 29.6)
Educational level achieved	No education	134,689 (31.1%)	26.1	(25.8, 26.4)
	Primary graduate	97,869 (22.6%)	30.5	(30.1, 30.9)
	Secondary graduate	159,998 (37.0%)	39.9	(39.6, 40.2)
	Higher than secondary	40,312 (9.3%)	42.6	(41.9, 43.3)
Women’s empowerment score (0–10): Mean (SD)		5.47 (1.98)		
Wealth quintile	Poorest	105,312 (24.3%)	31.5	(31.2, 31.9)
	Poorer	94,905 (21.9%)	33.2	(32.9, 33.6)
	Middle	85,933 (19.9%)	34.0	(33.6, 34.4)
	Richer	78,869 (18.2%)	34.8	(34.4, 35.3)
	Richest	67,849 (15.7%)	36.6	(36.0, 37.1)
Marital status	Not married	67,756 (15.7%)	28.4	(28.0, 28.9)
	Currently married	365,112 (84.3%)	34.9	(34.7, 35.1)
Residence place	Urban	121,133 (28.0%)	35.3	(34.9, 35.7)
	Rural	311,735 (72.0%)	33.3	(33.1, 33.5)
***Baby demographic factors***				
Sex of child	Male	225,816 (52.2%)	34.3	(34.0, 34.6)
	Female	207,052 (47.8%)	33.5	(33.2, 33.8)
Birth order	1	115,076 (26.6%)	37.5	(37.1, 37.9)
	2	112,654 (26.0%)	37.4	(37.0, 37.7)
	3	71,920 (16.6%)	33.8	(33.3, 34.2)
	≥4	133,218 (30.8%)	27.7	(27.4, 28.0)
***Known medical risk factors***				
History o neonatal mortality	No	408,472 (94.4%)	34.1	(33.9, 34.3)
	Yes	24,396 (5.6%)	30.4	(29.7, 31.1)
Multiple birth	No	426,715 (98.6%)	33.9	(33.8, 34.1)
	Yes	6,153 (1.4%)	32.0	(30.4, 33.5)
Cesarean section	No	376,740 (87.0%)	32.6	(32.4, 32.8)
	Yes	56,128 (13.0%)	41.8	(41.2, 42.4)
Being small or low birthweight or preterm birth of baby	No	327,112 (75.6%)	33.7	(33.5, 33.9)
	Yes	105,756 (24.4%)	34.7	(34.3, 35.1)
***Utilization of perinatal services***				
Number of ANC visits	<4	180,873 (41.9%)	26.6	(26.4, 26.9)
	≥4	250,526 (58.1%)	39.2	(38.9, 39.4)
First ANC visits	>3 months of pregnancy	206,357 (47.7%)	27.4	(27.1, 27.6)
	≤3 months of pregnancy	226,511 (52.3%)	39.8	(39.6, 40.1)
Delivery place	Home	103,506 (23.9%)	28.0	(27.6, 28.3)
	Institution	329,362 (76.1%)	35.6	(35.4, 35.9)
	Primary	184,869 (42.7%)	35.5	(35.3, 35.8)
	Public primary	138,981 (32.1%)	36.3	(36.0, 36.7)
	Private primary	46,171 (10.7%)	33.8	(33.2, 34.3)
	Secondary	144,493 (33.4%)	35.8	(35.4, 36.1)
	Public secondary	120,612 (27.9%)	33.7	(33.3, 34.1)
	Private secondary	23,598 (5.5%)	43.4	(42.6, 44.3)
Full utilization of perinatal services^**^	No	279,159 (64.7%)	30.0	(29.8, 30.2)
	Yes	152,240 (35.3%)	41.2	(40.8, 41.5)
***Quality of perinatal services***				
ANC quality score (0–10): Mean (SD, min–max)		8.09 (2.94, 0–10)		
IPN quality score (0–10): Mean (SD, min–max)		7.20 (3.27, 0–10)		
Composite quality score (0–10): Mean (SD, min–max)		7.88 (2.56, 0–10)		
Tertile of composite quality score				
1st tertile (low)		127,041 (29.4%)	18.5	(18.2, 18.8)
2nd tertile (medium)		153,581 (35.5%)	36.1	(35.8, 36.4)
3rd tertile (high)		152,246 (35.2%)	44.5	(44.2, 44.9)

**Full utilization of perinatal services was defined as attending four or more ANC visits, initiating ANC visit within the first trimester of pregnancy, and delivering in a health facility.

Abbreviations: ANC, antenatal care; CI, confidence interval; SD, standard deviation.

Within the analytical sample, 9.9% of mothers were older than 35 years and 12.0% were younger than 20 years at the time of pregnancy (Table 1). A total of 53.6% of mothers had received a primary education or less, and approximately 72.0% resided in rural areas. In terms of birth methods, 13.0% of the sample reported having given birth by Cesarean section and approximately 24.4% of respondents indicated that their babies were small in size, had low birth weight, or were born preterm.

The proportion of babies receiving postnatal checkup within 28 days of birth was lower among mothers who were younger than 20 years or older than 35 years at the time of pregnancy, compared with those aged 20–35 years. A higher proportion of postnatal checkups within 28 days was observed among babies born to mothers who were more educated, wealthier, married, residing in urban areas, delivered by Cesarean section, attended four or more ANC visits, initiated ANC visit within the first trimester, and delivered in a health facility, compared to their respective counterparts (Table 1).

The mean composite quality score of perinatal services was 7.88, ranging from 0 to 10 (Table 1). Mean scores varied by country (Table E in S1 Appendix). In general, countries with lower ANC quality scores also tended to have lower IPC quality scores. For example, Afghanistan, which reported the lowest ANC quality score also reported the second-lowest IPC quality score among the 38 countries. The average IPC score in Chad was only 1.12 (Table E in S1 Appendix).

**Fig 1:**
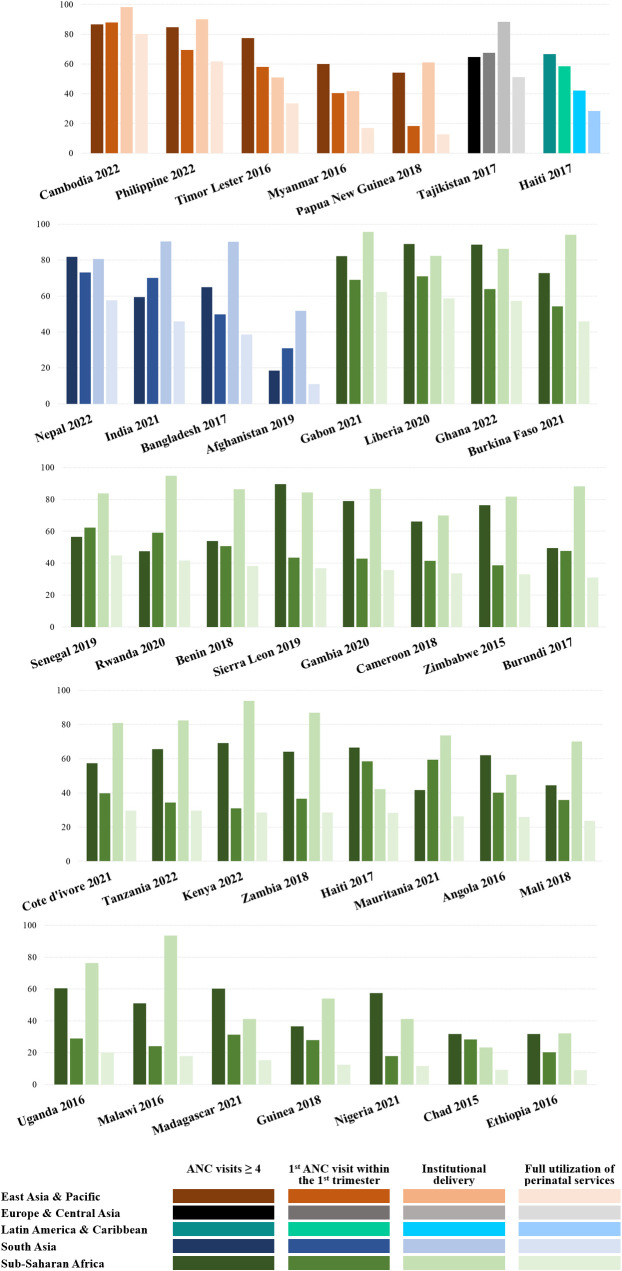
Perinatal care utilization rates across the 38 countries included in the analysis for: (i) ≥4 ANC visits; (ii) 1st ANC visit within the 1st trimester of pregnancy; (iii) institutional delivery; and (iv) full utilization of perinatal services.

A total of 35.3% of respondents in the analytic sample reported having fully utilized perinatal services. Fig 1 presents both individual and composite measures of perinatal care utilization rates across the 38 countries included in the analysis, sorted in descending order by full utilization rates. Full utilization of perinatal services varied considerably within and across regions, with the highest rates observed in East Asia—particularly Cambodia (80.0%)—and the lowest in Ethiopia (9.1%).

### Associations between utilization and quality of perinatal services and receipt of postnatal checkup within 28 days of birth

Full utilization of perinatal services—defined as attending four or more visits, initiating ANC visit within the first trimester, and delivering in a health facility—was associated with a higher likelihood of a baby receiving a postnatal check within 28 days (adjusted Odds Ratio [aOR] 1.27; 95% CI [1.26, 1.29]; *p* < 0.001) (Model 1 in Table 2). After additionally including the perinatal service quality, the association persisted, although the magnitude was attenuated (aOR 1.13; 95% CI [1.11,1.14]; *p* < 0.001) (Model 2 in Table 2).

**Table 2:** Association between utilization and quality of perinatal services and the likelihood of receiving a postnatal care within 28 days of birth.

	OR	(95% CI)	*p*-value	OR	(95% CI)	*p*-value	OR	(95% CI)	*p*-value
Variable	Model 1			Model 2			Model 3		
Full utilization of perinatal services (ref = no)									
Yes	1.27	(1.26, 1.29)	<0.001	1.13	(1.11, 1.14)	<0.001	1.00	(0.95, 1.05)	0.97
Tertiles of composite quality score (ref = low)									
Medium				1.92	(1.88, 1.96)	<0.001	1.90	(1.86, 1.95)	<0.001
High				2.59	(2.53, 2.65)	<0.001	2.49	(2.43, 2.55)	<0.001
Full utilization × composite quality score (ref = low)									
Medium							1.10	(1.05, 1.16)	<0.001
High							1.18	(1.12, 1.24)	<0.001

Model 1 includes full utilization of perinatal services only.

Model 2 additionally includes tertiles of the composite quality score.

Model 3 further includes the interaction between full utilization and the composite quality score.

All models are adjusted for maternal sociodemographic factors, baby demographic factors, and known medical risk factors.

Full utilization of perinatal services was defined as attending four or more ANC visits, initiating ANC visit within the first trimester of pregnancy, and delivering in a health facility.

Abbreviations: ANC, antenatal care; CI, confidence interval; OR, odds ratio.

Higher perinatal service quality received was also positively associated with receipt of a postnatal check within 28 days. Compared to babies born to mothers who received low-quality perinatal services, those whose mothers received medium- and high-quality services had higher odds of receiving a postnatal check (aOR 1.92; 95% CI [1.88–1.96] and aOR 2.59; 95% CI [2.53–2.65], respectively; *p* < 0.001 for both) (Model 2 in Table 2). Similar patterns were observed when the composite quality score was modeled as a continuous variable in sensitivity analyses (Model 2-1 in Table F in S1 Appendix)

**Fig 2:**
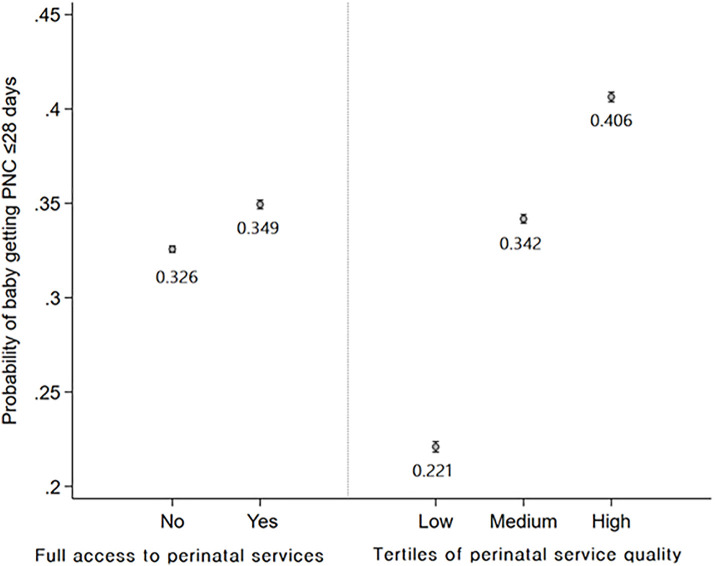
Probability of a baby getting PNC within 28 days based on full utilization of the perinatal services and tertiles of the composite quality score (postestimated from Model 2).

Predicted probabilities postestimated from model 2 indicated that babies born to mothers who fully utilized perinatal services had a higher probability of receiving a postnatal checkup within 28 days than those who did not (0.349 versus 0.326). However, the probability gap across tertiles of perinatal service quality was more pronounced with predicted probability ranging from 0.221 in the lowest quality tertile to 0.406 in the highest tertile (Fig 2).

The association between full utilization and receipt of a postnatal check within 28 days of birth varied across levels of perinatal service quality (Model 3 in Table 2 and Model 3-2 in Table F in S1 Appendix). Postestimation results showed that, among mothers in the lowest tertile of service quality, predicted probabilities of a baby receiving a postnatal check within 28 days were nearly identical regardless of full utilization status (0.217 versus 0.216). In contrast, among mothers in the highest quality tertile, the predicted probability increased from 0.392 among those without full utilization to 0.428 among those with full utilization (Fig A in S1 Appendix).

### Interactions between perinatal service quality and wealth level among mothers with full utilization of perinatal services

The association between perinatal service quality and receipt of PNC within 28 days of birth differed across wealth level (Model 4 in Table G in S1 Appendix). Specifically, compared with mothers in the poorest quintile (reference group), the positive association between high (vs. low) service quality and receipt of PNC appeared weaker among wealthier groups.

For example, the interaction between the 3rd wealth quintile and high quality tertile yielded an aOR of 0.85 (95% CI [0.74–0.99]; *p* = 0.036), indicating a smaller relative difference compared with the poorest quintile. This attenuation was more pronounced in the richest quintile (5th) (aOR 0.80; 95% CI [0.69–0.93]; *p* = 0.003). Results were consistent when service quality was modeled as a continuous variable (Model 4-1, Table G in S1 Appendix).

**Fig 3:**
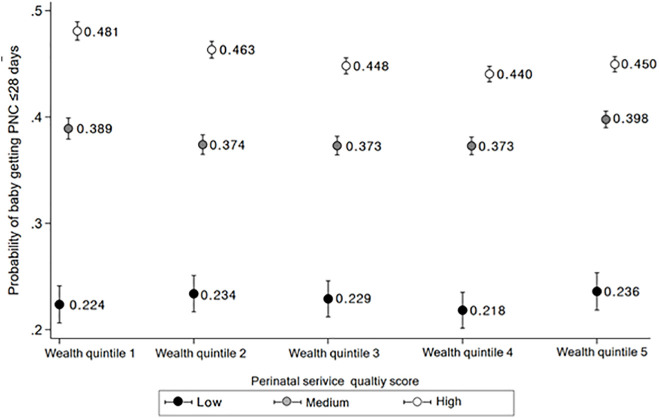
Probability of a baby getting PNC within 28 days according to tertile of composite quality of perinatal services (low-, medium-, and high-quality) in each of five wealth level among mothers with full utilization of the perinatal services (postestimated from Table H in S1 Appendix).

Predicted probabilities derived from wealth-stratified models further illustrated these patterns (Table H in S1 Appendix and Fig 3). Among mothers in the poorest wealth quintile, the predicted probability of a baby receiving postnatal checkup within 28 days increased from 0.224 in the low-quality group to 0.481 in the high-quality group. Among mothers in the richest wealth quintile, the corresponding probabilities were 0.236 and 0.450, respectively, reflecting a narrower difference across quality levels.

**Fig 4:**
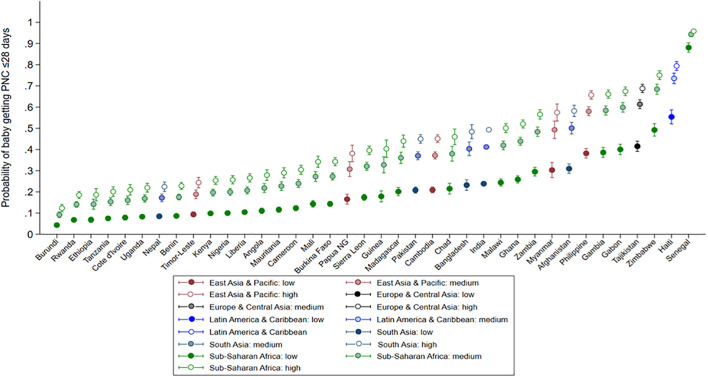
Probability of a baby receiving postnatal check within 28 days according to tertile of composite quality score among mothers who had full utilization of perinatal services in each of 38 countries.

When countries were ranked according to the predicted probability of postnatal checkup receipt within 28 days, those with higher overall predicted probability generally exhibited a larger absolute difference between the high- and low-quality groups. Predicted probability was lowest in Burundi and highest in Senegal (Fig 4 and Table I in S1 Appendix).

Among covariates, maternal education level, residence place, child’s sex, and all known medical risk factors for neonatal mortality were associated with the likelihood of a baby postnatal check within 28 days (Table J in S1 Appendix)

## Discussion

Our study demonstrates that high-quality perinatal care significantly increases the likelihood of PNC utilization within 28 days. Specifically, among mothers who fully utilized perinatal services, the odds of the baby receiving PNC within 28 days approximately twice as high among mothers who experienced high-quality perinatal care compared to those who received lower-quality care. This relationship was consistent across wealth quintiles (as shown in Fig 3). Furthermore, the impact of improved care quality was most pronounced among the lowest socioeconomic groups, suggesting that high-quality perinatal care may serve as an equity-promoting intervention, potentially narrowing the well-documented socioeconomic disparities in maternal healthcare utilization. This challenges the traditional narrative that focuses primarily on overcoming individual-level barriers to accessing care and instead points to the responsibility of health systems to deliver high-quality care that effectively engages all populations. Current strategies to improve PNC utilization have primarily focused on addressing access barriers and ensuring continuity of care throughout the perinatal period. The underlying assumption is that women who maintain regular contact with the healthcare system during pregnancy are more likely to return for PNC. However, despite these efforts, PNC uptake remains persistently low.

Our interaction analysis revealed a critical insight: high-quality care substantially enhanced the magnitude of the association of expanded perinatal service use with PNC use, whereas increased access alone was not linked to higher PNC utilization (as shown in Fig A in S1 Appendix).

While high-quality care has long been recognized as essential for achieving positive health outcomes, its role in increasing service utilization has been less emphasized [19–21]. Existing approaches to improving maternal and newborn health typically rest on an assumption that there are two parts to ensuring effective access to care: (i) improved access will promote more utilization, and (ii) improved quality will ameliorate health outcomes. However, our findings challenge this dichotomy, showing that care quality is important for both parts of the equation. We demonstrate that quality is not only vital for achieving better health outcomes but also plays a crucial role in determining whether women engage with postnatal services. This observation aligns with the Lancet Global Health Commission’s high-quality health system framework [22], which explains that when the process quality of care is high, women may gain greater confidence in the health system or perceive themselves to be in better health, which in turn can increase their health needs and lead to further service utilization (Fig B in S1 Appendix). This feedback loop may, in turn, reinforce improvements in process quality, creating a virtuous cycle.

Notably, the impact of improved care quality was most pronounced among the lowest wealth groups, highlighting its potential as a mechanism for promoting equity. Our analysis of users with full perinatal service utilization indicates that when care quality was low, the probability of PNC use was similar across wealth quintiles yet when perinatal care quality was high, the highest probability of PNC use was observed in the lowest wealth quintile. These findings diverge from much of the existing literature that suggests that lower wealth groups are typically less engaged with the health system [22,23]. In contrast, our results indicate that when provided with high-quality care, lower wealth groups have a higher probability of PNC use than wealthier groups showing that the benefit of raising care quality is most pronounced among the poor compared to wealthier groups.

Our results broaden the conventional understanding of the role of quality in maternal healthcare and highlight the structural determinants of PNC engagement. In particular, by showing that perinatal quality affects the likelihood of PNC use, our findings place accountability on health systems and policymakers to ensure high-quality care throughout the perinatal period. In doing so, our findings reinforce the need for systemic improvements, emphasizing that sustained PNC uptake depends not only on women’s choices but also on the quality and responsiveness of the healthcare services available to them.

Several limitations should be noted. First, due to constraints in data availability, our measure of ANC and IPC quality is based only on six and four indicators, respectively. Consequently, our quality indicator may not fully capture the entire spectrum of ANC and IPC quality. However, we believe this limitation does not compromise the validity of our findings. A prior study demonstrated an association between the composite quality index constructed in the same way as ours and neonatal mortality [24]. Second, the utilization and quality measures relied on maternal self-reports, which may be subject to recall bias. However, previous validation studies suggest that the accuracy of self-reported ANC and PNC content indicators is generally high, particularly for concrete, observable clinical activities (e.g., diagnostic tests), compared to less tangible components such as counseling content (e.g., discussion about HIV) [24,25]. Third, the DHS does not provide information on whether appropriate follow-up care was delivered based on blood or urine test results, which represents another limitation of our study. Finally, although we applied sampling weights when presenting descriptive statistics, our main regression analyses did not account for the complex DHS survey design, including sampling weights, strata, and clusters. Consequently, standard errors may be underestimated. However, prior methodological literature indicates that the use of unweighted models remains appropriate when the primary objective is to examine associations rather than population-level parameters. Therefore, we do not believe that this analytic choice is likely to compromise the validity of our substantive findings [26–29]. Lastly, the cross-sectional design precludes any inference of causality.

Despite limitations, our study has several notable strengths. We demonstrated that high- quality perinatal services shapes women’s likelihood of returning for postnatal services beyond improving newborn outcomes. We further demonstrated that these benefits of improving the quality of perinatal care are greater among lower-income groups compared to wealthier groups. These findings are based on the most recent, nationally representative household survey data, thereby enhancing the generalizability and policy relevance of our results.

Our findings have implications for current health policy approaches in LMICs, which often prioritize expanding access through initiatives such as increasing facility-based births or ANC coverage [30,31]. While these access-focused interventions are necessary, our results suggest they may be insufficient without concurrent quality improvements. Our findings call for a fundamental shift in how maternal health interventions are conceptualized and implemented. Rather than viewing quality as a secondary consideration to access, our results suggest that quality should be central to maternal health system strengthening efforts. This has several practical implications for policy and program design, highlighting the need to integrate quality metrics into maternal health program evaluation frameworks, the importance of investing in system-wide quality improvement initiatives alongside access expansion, and the potential for quality improvement as an equity-promoting strategy. This study adds to the growing evidence base supporting a shift from access-focused to quality-focused maternal health interventions. By demonstrating the central role of care quality in promoting PNC utilization, particularly among disadvantaged populations, our findings suggest that improving health system quality could be a more effective strategy for achieving universal maternal healthcare coverage than traditional access-focused approaches.

**A prespecified statistical analysis plan:** No formal prespecified statistical analysis plan was developed for this study. However, the analytical approach was guided by the study objectives and existing literature, and key variables and models were defined prior to data analysis.

## Supporting information

S1 STROBE Checklist The STROBE checklist is best used in conjunction with this article (freely available on the Web sites of PLoS Medicine at http://www.plosmedicine.org/, Annals of Internal Medicine at http://www.annals.org/, and Epidemiology at http://www.epidem.com/). Information on the STROBE Initiative is available at www.strobe-statement.org.
(DOCX)

S1 Appendix **Table A.** Comparison of process indicators for ANC and IPNC: WHO recommendations versus our study. **Table B.** Countries and survey years included in analytic sample. Table C. Comparison of original sample and final analytic sample. **Table D.** Comparison between original sample and final analytic sample of Bangladesh. **Table E.** ANC, IPC, and composite quality score by country. **Table F.** Association between utilization and quality of perinatal services and the likelihood of receiving a postnatal care within 28 days of birth, with service quality modeled as a continuous variable. **Table G.** Interactions between quality of perinatal services and wealth level among mothers who had fully utilized perinatal services. **Table H.** The association between the quality of perinatal services and baby receiving a postnatal check within 28 days from wealth-stratified analyses among mothers who had full utilization of perinatal services. **Table I.** Probability of a baby getting PNC within 28 days after birth and 95% CI in 38 countries. **Table J.** Association between covariates and the likelihood of a baby receiving a postnatal check within 28days (from Model 2). **Fig A.** Probability of babies receiving a postnatal check within 28 days when mother did and did not fully utilize perinatal services, across low (bottom 33.3%), medium (medium 33.3%) and high (top 33.3%) composite quality score group (postestimated from Model 3). **Fig B.** High-quality health system framework suggested by Lancet Global Health Commission (2018).
(DOCX)

## Abbreviations

ANC: antenatal care;
aOR: adjusted Odds Ratio;
CI: confidence interval
DHS: Demographic and Health Survey
IPC: immediate postpartum care;
LMICs: low- and middle-income countries;
PNC: postnatal care;
PSUs: primary sampling units;
SD: standard deviation;
SDG: Sustainable Development Goal
STROBE: Strengthening the Reporting of Observational Studies in Epidemiology;
WHO: World Health Organization.
