# Supplementary material for: Quality of antenatal and delivery care and postnatal care use: A multi-country observational study of 400,000 births
Source: PLoS Med. 2026 Apr 21;23(4):e1005055. doi: 10.1371/journal.pmed.1005055 (PMC13098959; doi:10.1371/journal.pmed.1005055)
Supplement: S1 STROBE Checklist — Information on the STROBE Initiative is available at www.strobe-statement.org. (DOCX) [file pmed.1005055.s001.docx]

STROBE Statement—Checklist of items that should be included in reports of ***cross-sectional studies***

|  | Item No | Recommendation | Page No |
| --- | --- | --- | --- |
| **Title and abstract** | 1 | (*a*) Indicate the study’s design with a commonly used term in the title or the abstract | The 2^nd^ paragraph in the Abstract section |
|  |  | (*b*) Provide in the abstract an informative and balanced summary of what was done and what was found | The 2^nd^ paragraph in the Abstract section |
| Introduction | | | |
| Background/rationale | 2 | Explain the scientific background and rationale for the investigation being reported | The second-to-last paragraph of the Introduction section |
| Objectives | 3 | State specific objectives, including any prespecified hypotheses | The last paragraph of the Introduction section |
| Methods | | | |
| Study design | 4 | Present key elements of study design early in the paper | The data source, settings and sample subsection of the Methods section |
| Setting | 5 | Describe the setting, locations, and relevant dates, including periods of recruitment, exposure, follow-up, and data collection | The data source, settings and sample subsection of the Methods section and Table S1 |
| Participants | 6 | (*a*) Give the eligibility criteria, and the sources and methods of selection of participants | The data source, settings and sample subsection of the Methods section |
| Variables | 7 | Clearly define all outcomes, exposures, predictors, potential confounders, and effect modifiers. Give diagnostic criteria, if applicable | The Outcome and main interest variables subsection and Covariates subsection of the Methods section |
| Data sources/ measurement | 8* | For each variable of interest, give sources of data and details of methods of assessment (measurement). Describe comparability of assessment methods if there is more than one group | The Outcome and main interest variables subsection and Covariates subsection of the Methods section |
| Bias | 9 | Describe any efforts to address potential sources of bias | The third- to- last paragraph of the Discussion section |
| Study size | 10 | Explain how the study size was arrived at | The first paragraph of the Analytic sample subsection of the Results section and Table S3 |
| Quantitative variables | 11 | Explain how quantitative variables were handled in the analyses. If applicable, describe which groupings were chosen and why | The Outcome and main interest variables subsection and Covariates subsection of the Methods section |
| Statistical methods | 12 | (*a*) Describe all statistical methods, including those used to control for confounding | The Statistical analysis subsection of the Methods section |
|  |  | (*b*) Describe any methods used to examine subgroups and interactions | The Statistical analysis subsection of the Methods section |
|  |  | (*c*) Explain how missing data were addressed | The last paragraph of the Statistical analysis subsection of the Methods section |
|  |  | (*d*) If applicable, describe analytical methods taking account of sampling strategy | The first paragraph of the Statistical analysis subsection of the Methods section |
|  |  | (*e*) Describe any sensitivity analyses | NA |
| Results | | | |
| Participants | 13* | (a) Report numbers of individuals at each stage of study—eg numbers potentially eligible, examined for eligibility, confirmed eligible, included in the study, completing follow-up, and analysed | The first paragraph of the Analytic sample subsection of the Results section and Table S3 |
|  |  | (b) Give reasons for non-participation at each stage | NA |
|  |  | (c) Consider use of a flow diagram | NA |
| Descriptive data | 14* | (a) Give characteristics of study participants (eg demographic, clinical, social) and information on exposures and potential confounders | S3 Table in supplemental material |
|  |  | (b) Indicate number of participants with missing data for each variable of interest | Table 1 |
| Outcome data | 15* | Report numbers of outcome events or summary measures | Table 1 |
| Main results | 16 | (*a*) Give unadjusted estimates and, if applicable, confounder-adjusted estimates and their precision (eg, 95% confidence interval). Make clear which confounders were adjusted for and why they were included | Table 2, \|Tabld S6 |
|  |  | (*b*) Report category boundaries when continuous variables were categorized | The Outcome and main interest variables subsection and Covariates subsection of the Methods section |
|  |  | (*c*) If relevant, consider translating estimates of relative risk into absolute risk for a meaningful time period | NA |
| Other analyses | 17 | Report other analyses done—eg analyses of subgroups and interactions, and sensitivity analyses | “Interactions between perinatal service quality and wealth level among mothers with full utilization of perinatal services” subsection of the Results section |
| Discussion | | | |
| Key results | 18 | Summarise key results with reference to study objectives | The first paragraph of the Discussion section |
| Limitations | 19 | Discuss limitations of the study, taking into account sources of potential bias or imprecision. Discuss both direction and magnitude of any potential bias | The third- to- last paragraph of the Discussion section |
| Interpretation | 20 | Give a cautious overall interpretation of results considering objectives, limitations, multiplicity of analyses, results from similar studies, and other relevant evidence | The third- to- last paragraph of the Discussion section |
| Generalisability | 21 | Discuss the generalisability (external validity) of the study results | The last paragraph of the Discussion section |
| Other information | | | |
| Funding | 22 | Give the source of funding and the role of the funders for the present study and, if applicable, for the original study on which the present article is based | “Funding statement” after Discussion section. |

*Give information separately for exposed and unexposed groups.

**Note:** An Explanation and Elaboration article discusses each checklist item and gives methodological background and published examples of transparent reporting. The STROBE checklist is best used in conjunction with this article (freely available on the Web sites of PLoS Medicine at http://www.plosmedicine.org/, Annals of Internal Medicine at http://www.annals.org/, and Epidemiology at http://www.epidem.com/). Information on the STROBE Initiative is available at www.strobe-statement.org.
