# Supplementary material for: Quality of antenatal and delivery care and postnatal care use: A multi-country observational study of 400,000 births
Source: PLoS Med. 2026 Apr 21;23(4):e1005055. doi: 10.1371/journal.pmed.1005055 (PMC13098959; doi:10.1371/journal.pmed.1005055)
Supplement: S1 Appendix — Table A. Comparison of process indicators for ANC and IPNC: WHO recommendations versus our study. Table B. Countries and survey years included in analytic sample. Table C. Comparison of original sample and final analytic sample. Table D. Comparison between original sample and final analytic sample of Bangladesh. Table E. ANC, IPC, and composite quality score by country. Table F. Association between utilization and quality of perinatal services and the likelihood of receiving a postnatal care within 28 days of birth, with service quality modeled as a continuous variable. Table G. Interactions between quality of perinatal services and wealth level among mothers who had fully utilized perinatal services. Table H. The association between the quality of perinatal services and baby receiving a postnatal check within 28 days from wealth-stratified analyses among mothers who had full utilization of perinatal services. Table I. Probability of a baby getting PNC within 28 days after birth and 95% CI in 38 countries. Table J. Association between covariates and the likelihood of a baby receiving a postnatal check within 28days (from Model 2). Fig A. Probability of babies receiving a postnatal check within 28 days when mother did and did not fully utilize perinatal services, across low (bottom 33.3%), medium (medium 33.3%) and high (top 33.3%) composite quality score group (postestimated from Model 3). Fig B. High-quality health system framework suggested by Lancet Global Health Commission (2018). (DOCX) [file pmed.1005055.s002.docx]

Table A. Comparison of process indicators for ANC and IPNC: WHO recommendations vs. our study^§^

| ANC | | PNC | |
| --- | --- | --- | --- |
| WHO | Inclusion | WHO | Inclusion |
| Dietary interventions | Indirectly ⭘ (Dietary intervention is to prevent hypertension and excessive weight gain. Instead, we included weight measurement, and BP check) | Assessment of baby | ⭘ (Examination within 24h after birth, examination by a doctor, nurse, or midwife) |
| Iron and folic acid supplements | ⭘ (Provision or purchase of iron supplements) | Exclusive breastfeeding and early initiation of breastfeeding within one hour of birth | ⭘ (Initiation of breastfeeding within one hour of birth)^**^ |
| Gestational diabetes mellitus | ⭘ (Urine test, blood test) | Daily chlorhexidine application | x |
| Tobacco use | x | Bathing delayed until 24 hours after birth | x |
| Substance use | x | Preterm and low-birth-weight babies should be identified immediately after birth | ⭘ (Newborn weight measurement after birth,) |
| HIV and syphilis testing | ⭘ (Blood test) | § We considered only the universally recommended indicators and excluded context-specific recommendations  ** Our indicator focuses on immediate postnatal care; therefore, **exclusive breastfeeding for six months was excluded**  † Only a very limited number of countries was examined | |
| Ultrasound scan | x ^†^ |  |  |
| Tetanus toxoid vaccination | ⭘ (Tetanus injection) |  |  |

Table B. Countries and survey years included in analytic sample

| No | Country | Survey year | No | Country | Survey year |
| --- | --- | --- | --- | --- | --- |
| 1 | Afghanistan | 2015 | 20 | Madagascar | 2021 |
| 2 | Angola | 2015-2016 | 21 | Malawi | 2015-2016 |
| 3 | Bangladesh | 2017-2018 | 22 | Mali | 2018 |
| 4 | Benin | 2017-2018 | 23 | Mauritania | 2019-2021 |
| 5 | Burundi | 2016-2017 | 24 | Myanmar | 2015-2016 |
| 6 | Burkina Faso | 2021 | 25 | Nepal | 2022 |
| 7 | Cambodia | 2021-2022 | 26 | Nigeria | 2021 |
| 8 | Cameroon | 2018 | 27 | Pakistan | 2017-2018 |
| 9 | Chad | 2014-2015 | 28 | Papua New Guinea | 2016-2018 |
| 10 | Cote d'Ivoire | 2021 | 29 | Philippine | 2022 |
| 11 | Ethiopia | 2016 | 30 | Rwanda | 2019-2020 |
| 12 | Gabon | 2019-2021 | 31 | Senegal | 2019 |
| 13 | Gambia | 2019-2020 | 32 | Sierra Leon | 2019 |
| 14 | Ghana | 2022 | 33 | Tanzania | 2022 |
| 15 | Guinea | 2018 | 34 | Tajikistan | 2017 |
| 16 | Haiti | 2016-2017 | 35 | Timor-Leste | 2016 |
| 17 | India | 2019-2021 | 36 | Uganda | 2016 |
| 18 | Kenya | 2022 | 37 | Zambia | 2018 |
| 19 | Liberia | 2019-2020 | 38 | Zimbabwe | 2015 |

Table C. Comparison of original sample and final analytic sample

| Variables | Categories | Original sample (N=457,084) | Final analytic sample  (N=432,868) |
| --- | --- | --- | --- |
| Maternal sociodemographic factors | | | |
| Pregnancy age | <20 | 54,951 (12.0%) | 52,003 (12.0%) |
|  | 20-35 | 356,522 (78.0%) | 338,218 (78.1%) |
|  | >35 | 45,611 (10.0%) | 42,647 (9.9%) |
| Educational level achieved | No education | 143,403 (31.4%) | 134,689 (31.1%) |
|  | Primary | 104,298 (22.8%) | 97,869 (22.6%) |
|  | Secondary | 167,490 (36.6%) | 159,998 (37.0%) |
|  | Higher | 41,892 (9.2%) | 40,312 (9.3%) |
|  | Missing | 1 (0.0%) | - |
| Wealth quintile | Poorest | 111,592 (24.4%) | 105,312 (24.3%) |
|  | Poorer | 100,097 (21.9%) | 94,905 (21.9%) |
|  | Middle | 90,557 (19.8%) | 85,933 (19.9%) |
|  | Richer | 83,338 (18.2%) | 78,869 (18.2%) |
|  | Richest | 71,500 (15.6%) | 67,849 (15.7%) |
| Marital status | Not married | 71,855 (15.7%) | 67,756 (15.7%) |
|  | Married | 385,229 (84.3%) | 365,112 (84.3%) |
| Residence place | Urban | 128,269 (28.1%) | 121,133 (28.0%) |
|  | Rural | 328,815 (71.9%) | 311,735 (72.0%) |
| Baby demographic factors |  |  |  |
| Sex of child | Male | 238,415 (52.2%) | 225,816 (52.2%) |
|  | Female | 218,669 (47.8%) | 207,052 (47.8%) |
| Birth order | 1 | 120,558 (26.4%) | 115,076 (26.6%) |
|  | 2 | 118,080 (25.8%) | 112,654 (26.0%) |
|  | 3 | 76,022 (16.6%) | 71,920 (16.6%) |
|  | ≥4 | 142,424 (31.2%) | 133,218 (30.8%) |
| Known medical risk factors | | | |
| History of neonatal mortality | No | 430,602 (94.2%) | 408,472 (94.4%) |
|  | Yes | 26,482 (5.8%) | 24,396 (5.6%) |
| Multiple birth | No | 444,144 (97.2%) | 426,715 (98.6%) |
|  | Yes | 12,940 (2.8%) | 6,153 (1.4%) |
| Caesarean section | No | 397,808 (87.0%) | 376,740 (87.0%) |
|  | Yes | 58,736 (12.9%) | 56,128 (13.0%) |
|  | Missing | 484 (0.1%) | - |
| Being small or low birthweight or preterm birth of baby | No | 340,573 (74.5%) | 327,112 (75.6%) |
|  | Yes | 112,389 (24.6%) | 105,756 (24.4%) |
|  | Missing | 4,122 (0.9%) | - |
| Utilization of perinatal services | | | |
| Number of ANC visits | <4 | 187,460 (41.1%) | 180,873 (41.9%) |
|  | ≥4 | 255,099 (56.0%) | 250,526 (58.1%) |
|  | Missing | 13,025 (2.9%) | - |
| First ANC visit | No visit or >3 months | 218,762 (47.9%) | 206,357 (47.7%) |
|  | ≤3 months | 236,065 (51.6%) | 226,511 (52.3%) |
|  | Missing | 2,257 (0.5%) | - |
| Full utilization of ANC services | No | 259,640 (59.2%) | 261,732 (60.7%) |
|  | Yes | 171,894 (37.7%) | 169,667 (39.3%) |
|  | Missing | 14,050 (3.1%) | - |
| Delivery place | Home | 111,238 (24.5%) | 103,506 (23.9%) |
|  | Institution | 342,314 (75.5%) | 329,362 (76.1%) |
|  | Primary | 191,007 (41.8%) | 184,869 (42.7%) |
|  | Public primary | 143,359 (31.4%) | 138,981 (32.1%) |
|  | Private primary | 47,955 (10.5%) | 46,171 (10.7%) |
|  | Secondary | 151,307 (33.1%) | 144,493 (33.4%) |
|  | Public secondary | 126,186 (27.6%) | 120,612 (27.9%) |
|  | Private secondary | 24,814 (5.4%) | 23,598 (5.5%) |
|  | Missing | 3,532 (0.8%) | - |
| Full utilization of perinatal services | No | 285,261 (62.6%) | 283,895 (65.6%) |
|  | Yes | 153,059 (33.6%) | 148,973 (34.4%) |
|  | Missing | 17,263 (3.8%) | - |
| Country and year |  |  |  |
| Afghanistan 2019 | | 17,979 | 17,979 |
| Missing | | 2,052 (10.2%) | - |
| Angola 2016 | | 8,572 | 8,572 |
| Missing | | 539 (5.9%) | - |
| Bangladesh 2017 | | 2,234 | 2,268 |
| Missing | | 2,832 (55.9%) | - |
| Benin 2018 | | 8,543 | 8,543 |
| Missing | | 703 (7.6%) | - |
| Burundi 2017 | | 8,195 | 8,195 |
| Missing | | 599 (6.8%) | - |
| Burkina Faso 2021 | | 6,357 | 6,357 |
| Missing | | 215 (3.3%) | - |
| Cambodia 2022 | | 4,618 | 4,618 |
| Missing | | 78 (1.7%) | - |
| Cameroon 2018 | | 6,212 | 6,212 |
| Missing | | 414 (6.2%) | - |
| Chad 2015 | | 10,702 | 10,702 |
| Missing | | 569 (5.0%) | - |
| Cote d’ivore 2021 | | 5,581 | 5,581 |
| Missing | | 215 (3.7%) | - |
| Ethiopia 2016 | | 7,041 | 7,041 |
| Missing | | 263 (3.6%) | - |
| Gabon 2021 | | 4,148 | 4,148 |
| Missing | | 415 (9.1%) | - |
| Gambia 2020 | | 5,626 | 5,626 |
| Missing | | 280 (4.7%) | - |
| Ghana 2022 | | 5,145 | 5,145 |
| Missing | | 217 (4.0%) | - |
| Guinea 2018 | | 5,221 | 5,221 |
| Missing | | 452 (8.0%) | - |
| Haiti 2017 | | 4,903 | 4,903 |
| Missing | | 203 (4.0%) | - |
| India 2021 | | 173,776 | 173,776 |
| Missing | | 4,722 (2.6%) | - |
| Kenya 2022 | | 9,404 | 9,404 |
| Missing | | 1,184 (11.2%) | - |
| Liberia 2020 | | 4,139 | 4,139 |
| Missing | | 216 (5.0%) | - |
| Madagascar 2021 | | 9,170 | 9,170 |
| Missing | | 244 (2.6%) | - |
| Malawi 2016 | | 13,078 | 13,078 |
| Missing | | 659 (4.8%) | - |
| Mali 2018 | | 6,150 | 6,150 |
| Missing | | 337 (5.2%) | - |
| Mauritania 2021 | | 6,955 | 6,955 |
| Missing | | 835 (10.7%) | - |
| Myanmar 2016 | | 3,641 | 3,641 |
| Missing | | 267(6.8%) | - |
| Nepal 2022 | | 2,856 | 2,856 |
| Missing | | 104(3.5%) | - |
| Nigeria 2021 | | 20,997 | 20,997 |
| Missing | | 1,244 (5.9%) | - |
| Pakistan 2018 | | 8,149 | 8,149 |
| Missing | | 264 (3.1%) | - |
| Papua New Guinea 2018 | | 5,728 | 5,728 |
| Missing | | 1,014 (15.0%) | - |
| Philippine 2022 | | 4,387 | 4,387 |
| Missing | | 101 (2.3%) | - |
| Rwanda 2020 | | 6,067 | 6,067 |
| Missing | | 206 (3.3%) | - |
| Senegal 2019 | | 4,170 | 4,170 |
| Missing | | 270 (6.1%) | - |
| Sierra Leon 2019 | | 6,498 | 6,498 |
| Missing | | 1,057 (14.0%) | - |
| Tanzania 2022 | | 5,710 | 5,710 |
| Missing | | 209 (3.5%) | - |
| Tajikistan 2017 | | 4,170 | 4,170 |
| Missing | | 125 (2.9%) | - |
| Timor Lester 2016 | | 4,825 | 4,825 |
| Missing | | 155 (3.1%) | - |
| Uganda 2016 | | 9,998 | 9,998 |
| Missing | | 465 (4.4%) | - |
| Zambia 2018 | | 7,201 | 7,201 |
| Missing | | 293 (3.9%) | - |
| Zimbabwe 2015 | | 4,688 | 4,688 |
| Missing | | 232 (4.7%) | - |

Table D. Comparison between original sample and final analytic sample of Bangladesh.

| Variables | Categories | Original sample (N=5,066) | Final analytic sample (N=2,268) |
| --- | --- | --- | --- |
| Maternal sociodemographic factors | | | |
| Pregnancy age | <20 | 1,453 (28.7%) | 667 (29.4%) |
|  | 20-35 | 3,488 (68.8%) | 1,546 (68.1%) |
|  | >35 | 127 (2.5%) | 57 (2.5%) |
| Educational level achieved | No education | 322 (6.3%) | 70 (3.1%) |
|  | Primary | 1,396 (27.5%) | 366 (16.1%) |
|  | Secondary | 2,482 (49.0%) | 1,184 (52.2%) |
|  | Higher | 869 (17.1%) | 651 (28.7%) |
| Women’s empowerment score (0~10): Mean (SD) | | 6.82 (1.34) | 7.02 (1.28) |
| Wealth quintile | Poorest | 1,044 (20.6%) | 249 (10.9%) |
|  | Poorer | 1,040 (20.5%) | 327 (14.4%) |
|  | Middle | 968 (19.1%) | 429 (18.9%) |
|  | Richer | 1021 (20.1%) | 543 (23.9%) |
|  | Richest | 995 (19.6%) | 722 (31.8%) |
| Marital status | Not married | 64 (1.3%) | 23 (1.0%) |
|  | Married | 5,003 (98.7%) | 2,246 (99.0%) |
| Residence place | Urban | 1,364 (26.9%) | 784 (34.6%) |
|  | Rural | 3,703 (73.1%) | 1,485 (65.4%) |
| Baby demographic factors |  |  |  |
| Sex of child | Male | 2,647 (52.2%) | 1,214 (53.5%) |
|  | Female | 2,420 (47.8%) | 1,055 (46.5%) |
| Birth order | 1 | 1,932 (38.1%) | 1,082 (47.7%) |
|  | 2 | 1,663 (32.8%) | 744 (32.8%) |
|  | 3 | 851 (16.8%) | 306 (13.5%) |
|  | ≥4 | 622 (12.3%) | 138 (6.1%) |
| Known medical risk factors | | | |
| History of neonatal mortality | No | 4,731 (93.4%) | 2,154 (95.0%) |
|  | Yes | 336 (6.6%) | 115 (5.0%) |
| Multiple birth | No | 4,962 (97.9%) | 2,240 (98.7%) |
|  | Yes | 105 (2.1%) | 29 (1.3%) |
| Caesarean section | No | 3,371 (66.5%) | 842 (37.1%) |
|  | Yes | 1,689 (33.3%) | 1,427 (62.9%) |
|  | Missing | 7 (0.1%) | - |
| Being small or low birthweight or preterm birth of baby | No | 1,941 (38.3%) | 1,919 (84.6%) |
|  | Yes | 375 (7.4%) | 350 (15.4%) |
|  | Missing | 2,752 (54.3%) | - |
| Utilization of perinatal services | | | |
| Number of ANC visits | <4 | 2,657 (52.4%) | 796 (35.1%) |
|  | ≥4 | 2,357 (46.5%) | 1,473 (64.9%) |
|  | Missing | 53 (1.0%) |  |
| First ANC visit | No visit or >3 months | 3,179 (62.7%) | 1141 (50.3%) |
|  | ≤3 months | 1,887 (37.2%) | 1128 (49.7%) |
|  | Missing | 1 (0.0%) | - |
| Full utilization of ANC services | No | 3,620 (71.5%) | 1,329 (58.6%) |
|  | Yes | 1,393 (27.5%) | 940 (41.4%) |
|  | Missing | 54 (1.1%) |  |
| Delivery place | Home | 2,509 (49.5%) | 222 (9.7%) |
|  | Institution | 2,541 (50.1%) | 2,047 (90.3%) |
|  | Primary | 1,763 (34.8%) | 1,416 (62.4%) |
|  | Public primary | 414 (8.2%) | 289 (12.7%) |
|  | Private primary | 1,349 (26.6%) | 1,128 (49.7%) |
|  | Secondary | 778 (15.4%) | 632 (27.8%) |
|  | Public secondary | 329 (6.5%) | 258 (11.3%) |
|  | Private secondary | 450 (8.9%) | 374 (16.5%) |
|  | Missing | 18 (0.3%) | - |
| Full utilization of perinatal services | No | 4,018 (79.3%) | 1,395 (61.5%) |
|  | Yes | 979 (19.3%) | 874 (38.5%) |
|  | Missing | 70 (1.4%) |  |
| ***Quality of perinatal services: Mean (SD)*** | |  |  |
| ANC quality score (0~10) | | 7.60 (3.00) | 8.75 (2.1) |
| Immediate PNC quality score (0~10) | | 6.91 (2.86) | 9.25 (1.31) |
| Composite quality score (0~10) | | 7.50 (2.32) | 8.95 (1.36) |

Table E. ANC, IPC, and composite quality score by country

| Countries*** | ANC quality score | IPC quality score | Composite quality score |
| --- | --- | --- | --- |
|  | Mean (SD) | Mean (SD) | Mean (SD) |
| All countries | 8.09 (2.94) | 7.20 (3.27) | 7.88 (2.56) |
| Afghanistan 2019 | **3.58 (3.33)** | **2.93 (2.24)** | **3.38 (2.24)** |
| Angola 2016 | **6.80 (3.92)** | **5.47 (3.17)** | **6.38 (2.95)** |
| Bangladesh 2017 | 8.43 (1.95) | 9.25 (1.28) | 8.99 (1.30) |
| Benin 2018 | **7.78 (2.94)** | **7.12 (3.03)** | 7.98 (2.41) |
| Burundi 2017 | **5.38 (2.50)** | **7.20 (2.67)** | **6.18 (2.01)** |
| Burkina Faso 2021 | 9.39 (1.27) | 8.06 (2.46) | 8.93 (1.19) |
| Cambodia 2022 | 8.81 (1.77) | 8.36 (2.45) | 8.66 (1.53) |
| Cameroon 2018 | 8.32 (3.18) | **6.86 (3.35)** | 7.93 (2.62) |
| Chad 2015 | **4.64 (4.05)** | **1.12 (1.41)** | **3.31 (2.54**) |
| Cote d’lvoire 2021 | 8.45 (2.65) | **7.02 (3.29)** | 8.00 (2.18) |
| Ethiopia 2016 | **5.29 (4.01)** | **4.63 (2.83)** | **5.20 (2.84)** |
| Gabon 2021 | 9.04 (2.17) | 8.56 (2.18) | 8.95 (1.62) |
| Gambia 2020 | 9.53 (1.16) | 8.05 (2.65) | 9.10 (1.09) |
| Ghana 2022 | 9.35 (1.62) | 8.31 (2.63) | 9.01 (1.44) |
| Guinea 2018 | **7.65 (3.47)** | **5.47 (3.81)** | **6.99 (2.98)** |
| Haiti 2017 | 8.34 (2.81) | **5.36 (3.31)** | **7.57 (2.31)** |
| India 2021 | 9.12 (1.74) | 8.46 (2.42) | 8.99 (1.57) |
| Kenya 2022 | 9.19 (1.85) | 8.36 (2.88) | 8.93 (1.66) |
| Liberia 2020 | 9.31 (1.73) | 7.79 (2.83) | 8.77 (1.63) |
| Madagascar 2021 | **5.84 (3.17)** | **5.84 (3.34)** | **6.00 (2.62)** |
| Malawi 2016 | 8.11 (1.69) | 7.74 (2.67) | 7.99 (1.57) |
| Mali 2018 | **7.03 (3.91)** | **6.30 (3.38)** | **6.98 (2.99)** |
| Mauritania 2021 | **7.78 (3.07)** | **5.73 (3.39)** | **7.15 (2.46)** |
| Myanmar 2016 | **7.54 (3.00)** | **6.68 (3.47)** | **7.30 (2.56)** |
| Nepal 2022 | 9.14 (1.82) | 7.57 (2.86) | 8.63 (1.65) |
| Nigeria 2021 | **6.89 (4.10)** | **4.61 (3.56)** | **6.25 (3.18)** |
| Pakistan 2018 | **6.71 (3.48)** | **4.93 (3.37)** | **6.09 (2.81)** |
| Papua New Guinea 2018 | **6.61 (3.66)** | **6.82 (3.19)** | **6.83 (2.86)** |
| Philippine 2022 | 8.70 (2.35) | 8.41 (2.49) | 8.63 (1.96) |
| Rwanda 2020 | 8.48 (2.14) | 8.55 (2.29) | 8.54 (1.64) |
| Senegal 2019 | 9.08 (2.06) | 7.26 (3.05) | 8.46 (1.77) |
| Sierra Leon 2019 | 9.32 (1.54) | 8.68 (2.26) | 9.10 (1.33) |
| Tanzania 2022 | 8.10 (2.41) | **6.85 (2.85)** | **7.57 (1.97)** |
| Tajikistan 2017 | **8.08 (2.58)** | 8.60 (2.09) | 8.47 (1.57) |
| Timor Lester 2016 | **7.16 (2.87)** | **6.74 (2.69)** | **7.03 (2.20)** |
| Uganda 2016 | **7.72 (2.14)** | **6.98 (3.06)** | **7.52 (1.92)** |
| Zambia 2018 | 8.72 (1.77) | 8.03 (2.75) | 8.52 (1.63) |
| Zimbabwe 2015 | 8.44 (2.36) | 8.13 (2.68) | 8.38 (1.96) |

Table F. Association between utilization and quality of perinatal services and the likelihood of receiving postnatal care within 28 days of birth, with service quality modeled as a continuous variable

|  | Model 2-1 | | | | Model 3-1 | | | |
| --- | --- | --- | --- | --- | --- | --- | --- | --- |
|  | OR (95% CI) *p-value* | | | | OR (95% CI) *p-value* | | | |
| Full utilization of perinatal service (ref=no) |  |  |  |  |  |  |  |  |
| Yes | 1.09 | (1.08, | 1.11) | <0.001 | 0.47 | (0.43, | 0.52) | <0.001 |
| Composite quality (0-10) | 1.24 | (1.24, | 1.25) | <0.001 | 1.23 | (1.22, | 1.23) | <0.001 |
| Full utilization of perinatal service x composite quality (0-10) |  |  |  |  | 1.10 | (1.09, | 1.11) | <0.001 |

- Model 2-1 corresponds to Model 2 in the main manuscript, with tertiles of quality replaced by the continuous composite quality score (0-10)
- Model 3-1 corresponds to Model 3, additionally including the interaction between full utilization and the continuous quality score.
- All models are adjusted for maternal sociodemographic factors, baby demographic factors, and known medical risk factors
- Full utilization of perinatal services was defined as attending four or more ANC visits, initiating ANC visit within the first trimester of pregnancy, and delivering in a health facility.

Table G. Interactions between quality of perinatal services and wealth level among mothers who had fully utilized perinatal services

|  | Model 4 | | | | | Model 4-1 | | | |
| --- | --- | --- | --- | --- | --- | --- | --- | --- | --- |
| Variables | OR | (95% CI) *p-value* | | | | OR | (95% CI) *p-value* | | |
| Wealth level (ref=1^st^ quintile, poorest) |  |  |  | |  |  |  |  |  |
| 2^nd^ | 1.05 | (0.91, | 1.20) | 0.505 | | 1.28 | (0.94, | 1.75) | 0.123 |
| 3^rd^ | 1.06 | (0.92, | 1.21) | 0.451 | | 1.41 | (1.03, | 1.93) | 0.030 |
| 4^th^ | 1.05 | (0.91, | 1.21) | 0.516 | | 1.46 | (1.07, | 1.99) | 0.016 |
| 5^th^ (richest) | 1.26 | (1.09, | 1.45) | 0.001 | | 2.15 | (1.59, | 2.91) | 0.000 |
| Composite quality score (0~10) |  |  |  | |  | 1.42 | (1.38, | 1.45) | <0.001 |
| Tertile of composite quality score (ref=low) |  |  |  | |  |  |  |  |  |
| Medium | 2.32 | (2.07, | 2.59) | | <0.001 |  |  |  |  |
| High | 3.55 | (3.18, | 3.95) | | <0.001 |  |  |  |  |
| Wealth level (ref=1^st^, poorest) x composite quality score (0 -10) |  |  |  | |  |  |  |  |  |
| 2^nd^ |  |  |  | |  | 0.97 | (0.94, | 1.00) | 0.059 |
| 3^rd^ |  |  |  | |  | 0.96 | (0.92, | 0.99) | 0.008 |
| 4^th^ |  |  |  | |  | 0.95 | (0.92, | 0.98) | 0.005 |
| 5^th^ (richest) |  |  |  | |  | 0.93 | (0.90, | 0.96) | <0.001 |
| Wealth level (ref=1^st^) x composite quality score (ref= low) |  |  |  | |  |  |  |  |  |
| 2^nd^ x medium | 0.92 | (0.79, | 1.07) | | 0.256 |  |  |  |  |
| 2^nd^ x high | 0.89 | (0.77, | 1.04) | | 0.134 |  |  |  |  |
| 3^rd^ x medium | 0.92 | (0.79, | 1.07) | | 0.3 |  |  |  |  |
| 3^rd^ x high | 0.85 | (0.74, | 0.99) | | 0.036 |  |  |  |  |
| 4^th^ x medium | 0.98 | (0.84, | 1.14) | | 0.748 |  |  |  |  |
| 4^th^ x high | 0.86 | (0.74, | 1.00) | | 0.046 |  |  |  |  |
| 5^th^ (richest) x medium | 0.99 | (0.85, | 1.15) | | 0.859 |  |  |  |  |
| 5^th^ (richest) x high | 0.80 | (0.69, | 0.93) | | 0.003 |  |  |  |  |

- All models are adjusted for maternal sociodemographic factors, baby demographic factors, and known medical risk factors
- Model 4 includes interaction terms between tertiles of perinatal service quality and wealth level
- Model 4-1 replicates Model 4 using the continuous composite quality score (0-10).

Table H. The association between the quality of perinatal services and baby receiving a postnatal check within 28 days from wealth-stratified analyses among mothers who had full utilization of perinatal services

|  | 1^st^ quintile  (N=23,807) | | | | 2^nd^ quintile  (N=27,971) | | | | 3^rd^ quintile  (N=30,250) | | | | 4^th^ quintile  (N=32,421) | | | | 5^th^ quintile  (N=34,524) | | | |
| --- | --- | --- | --- | --- | --- | --- | --- | --- | --- | --- | --- | --- | --- | --- | --- | --- | --- | --- | --- | --- |
|  | OR | (95% CI) p value | | | OR | (95% CI) p value | | | OR | (95% CI) p value | | | OR | (95% CI) p value | | | OR | (95% CI) p value | | |
| Tertile of composite quality score (ref=low) | | | | | | | | | | | | | | | | | | | | |
| Medium | 2.35 | (2.09, | 2.64) | <0.001 | 2.07 | (1.85, | 2.31) | <0.001 | 2.13 | (1.91, | 2.38) | <0.001 | 2.28 | (2.04, | 2.56) | <0.001 | 2.30 | (2.06, | 2.58) | <0.001 |
| High | 3.54 | (3.15, | 3.97) | <0.001 | 3.08 | (2.76, | 3.44) | <0.001 | 2.99 | (2.68, | 3.35) | <0.001 | 3.10 | (2.77, | 3.48) | <0.001 | 2.91 | (2.60, | 3.27) | <0.001 |

Table I. Probability of baby getting PNC within 28 days after birth and 95% CI in 38 countries

|  | Low quality | | | Medium quality | | | High quality | | |
| --- | --- | --- | --- | --- | --- | --- | --- | --- | --- |
| Variables | OR | (95% CI) | | OR | (95% CI) | | OR | (95% CI) | |
| Burundi | 0.043 | (0.037, | 0.050) | 0.092 | (0.079, | 0.105) | 0.123 | (0.106, | 0.141) |
| Rwanda | 0.068 | (0.061, | 0.076) | 0.141 | (0.127, | 0.154) | 0.185 | (0.169, | 0.202) |
| Ethiopia | 0.069 | (0.056, | 0.081) | 0.142 | (0.118, | 0.165) | 0.186 | (0.157, | 0.216) |
| Tanzania | 0.075 | (0.065, | 0.085) | 0.154 | (0.135, | 0.172) | 0.201 | (0.179, | 0.224) |
| Cote d'lvoire | 0.079 | (0.067, | 0.090) | 0.160 | (0.140, | 0.180) | 0.210 | (0.185, | 0.234) |
| Uganda | 0.083 | (0.073, | 0.093) | 0.169 | (0.152, | 0.186) | 0.220 | (0.199, | 0.241) |
| Nepal | 0.085 | (0.074, | 0.095) | 0.172 | (0.154, | 0.190) | 0.224 | (0.202, | 0.246) |
| Benin | 0.087 | (0.079, | 0.095) | 0.175 | (0.162, | 0.189) | 0.228 | (0.212, | 0.244) |
| Timor Leste | 0.094 | (0.082, | 0.106) | 0.188 | (0.168, | 0.209) | 0.244 | (0.219, | 0.269) |
| Kenya | 0.099 | (0.089, | 0.108) | 0.197 | (0.181, | 0.213) | 0.254 | (0.235, | 0.272) |
| Nigeria | 0.100 | (0.090, | 0.110) | 0.199 | (0.183, | 0.216) | 0.257 | (0.237, | 0.276) |
| Liberia | 0.104 | (0.094, | 0.115) | 0.207 | (0.190, | 0.224) | 0.266 | (0.246, | 0.286) |
| Angola | 0.111 | (0.098, | 0.124) | 0.218 | (0.197, | 0.239) | 0.279 | (0.254, | 0.304) |
| Mauritania | 0.116 | (0.104, | 0.129) | 0.227 | (0.207, | 0.248) | 0.290 | (0.266, | 0.314) |
| Cameroon | 0.123 | (0.111, | 0.135) | 0.240 | (0.221, | 0.258) | 0.304 | (0.283, | 0.326) |
| Mali | 0.143 | (0.128, | 0.159) | 0.272 | (0.249, | 0.296) | 0.342 | (0.316, | 0.368) |
| Burkina Faso | 0.144 | (0.132, | 0.155) | 0.273 | (0.256, | 0.290) | 0.343 | (0.324, | 0.362) |
| Papua New Guinea | 0.166 | (0.142, | 0.189) | 0.307 | (0.272, | 0.343) | 0.381 | (0.342, | 0.420) |
| Sierra Leon | 0.174 | (0.160, | 0.188) | 0.321 | (0.302, | 0.339) | 0.396 | (0.376, | 0.416) |
| Guinea | 0.179 | (0.153, | 0.205) | 0.328 | (0.290, | 0.365) | 0.403 | (0.362, | 0.444) |
| Madagascar | 0.201 | (0.182, | 0.221) | 0.361 | (0.334, | 0.387) | 0.439 | (0.410, | 0.468) |
| Pakistan | 0.208 | (0.194, | 0.223) | 0.370 | (0.352, | 0.389) | 0.450 | (0.430, | 0.470) |
| Cambodia | 0.209 | (0.196, | 0.223) | 0.372 | (0.355, | 0.389) | 0.451 | (0.433, | 0.469) |
| Chad | 0.215 | (0.190, | 0.241) | 0.380 | (0.345, | 0.415) | 0.460 | (0.423, | 0.497) |
| Bangladesh | 0.232 | (0.207, | 0.257) | 0.403 | (0.371, | 0.435) | 0.484 | (0.451, | 0.517) |
| India | 0.239 | (0.229, | 0.248) | 0.412 | (0.406, | 0.418) | 0.493 | (0.488, | 0.498) |
| Malawi | 0.244 | (0.227, | 0.261) | 0.419 | (0.399, | 0.440) | 0.501 | (0.479, | 0.522) |
| Ghana | 0.259 | (0.242, | 0.276) | 0.439 | (0.420, | 0.457) | 0.520 | (0.502, | 0.539) |
| Zambia | 0.295 | (0.275, | 0.316) | 0.484 | (0.462, | 0.506) | 0.565 | (0.543, | 0.587) |
| Myanmar | 0.303 | (0.267, | 0.339) | 0.493 | (0.452, | 0.534) | 0.574 | (0.534, | 0.615) |
| Afghanistan | 0.310 | (0.288, | 0.332) | 0.501 | (0.474, | 0.528) | 0.582 | (0.555, | 0.609) |
| Philippine | 0.382 | (0.359, | 0.405) | 0.580 | (0.559, | 0.601) | 0.657 | (0.638, | 0.677) |
| Gambia | 0.386 | (0.362, | 0.409) | 0.584 | (0.563, | 0.605) | 0.661 | (0.642, | 0.681) |
| Gabon | 0.400 | (0.375, | 0.425) | 0.599 | (0.576, | 0.621) | 0.674 | (0.654, | 0.695) |
| Tajikistan | 0.415 | (0.391, | 0.440) | 0.614 | (0.592, | 0.635) | 0.688 | (0.668, | 0.707) |
| Zimbabwe | 0.492 | (0.463, | 0.522) | 0.684 | (0.661, | 0.708) | 0.751 | (0.730, | 0.771) |
| Haiti | 0.554 | (0.521, | 0.587) | 0.735 | (0.710, | 0.760) | 0.794 | (0.773, | 0.815) |
| Senegal | 0.881 | (0.859, | 0.903) | 0.943 | (0.932, | 0.954) | 0.958 | (0.950, | 0.967) |

Table J . Association between covariates and the likelihood of baby receiving postnatal check within 28days (from Model 2)

| Classification | Variables | Categories | OR | (95% CI) p value | | | |
| --- | --- | --- | --- | --- | --- | --- | --- |
| **Maternal sociodemographic factors** | Pregnancy age | <20 | Ref | | | | |
|  |  | 20-35 | 1.00 | (0.98, | 1.03) | 0.773 | |
|  |  | >35 | 0.96 | (0.92, | 0.99) | 0.016 | |
|  | Educational level achieved | No education | Ref | | | |  |
|  |  | Primary | 1.07 | (1.05, | 1.09) | 0.000 | |
|  |  | Secondary | 1.02 | (1.00, | 1.04) | 0.044 | |
|  |  | Higher | 1.05 | (1.01, | 1.08) | 0.004 | |
|  | Wealth quintile | Poorest | Ref | | | |  |
|  |  | Poorer | 0.99 | (0.97, | 1.01) | 0.581 | |
|  |  | Middle | 1.01 | (0.99, | 1.03) | 0.441 | |
|  |  | Richer | 1.03 | (1.00, | 1.05) | 0.024 | |
|  |  | Richest | 1.15 | (1.12, | 1.18) | <0.001 | |
|  | Marital status | Not married | Ref | | | |  |
|  |  | Married | 1.00 | (0.98, | 1.03) | 0.953 | |
|  | Residence place | Urban | Ref | | | |  |
|  |  | Rural | 1.14 | (1.12, | 1.16) | <0.001 | |
|  | Women’s empowerment |  | 1.03 | (1.03, | 1.04) | <0.001 | |
| **Baby demographic factors** | Sex of child | Male | Ref | | | |  |
|  |  | Female | 0.97 | (0.96, | 0.99) | <0.001 | |
|  | Birth order | 1 | Ref | | | |  |
|  |  | 2 | 1.01 | (0.99, | 1.03) | 0.316 | |
|  |  | 3 | 1.01 | (0.97, | 1.03) | 0.366 | |
|  |  | ≥ 4 | 1.02 | (0.99, | 1.04) | 0.204 | |
| **Known medical risk factors** | History of neonatal mortality | No | Ref | | | |  |
|  |  | Yes | 1.05 | (1.02, | 1.08) | 0.003 | |
|  | Multiple birth | No | Ref | | | |  |
|  |  | Yes | 0.92 | (0.87, | 0.98) | 0.010 | |
|  | Caesarean section | No | Ref | | | |  |
|  |  | Yes | 1.09 | (1.07, | 1.11) | <0.001 | |
|  | Baby small or low birthweight or preterm birth | No | Ref | | | |  |
|  |  | Yes | 1.03 | (1.01, | 1.04) | 0.001 | |


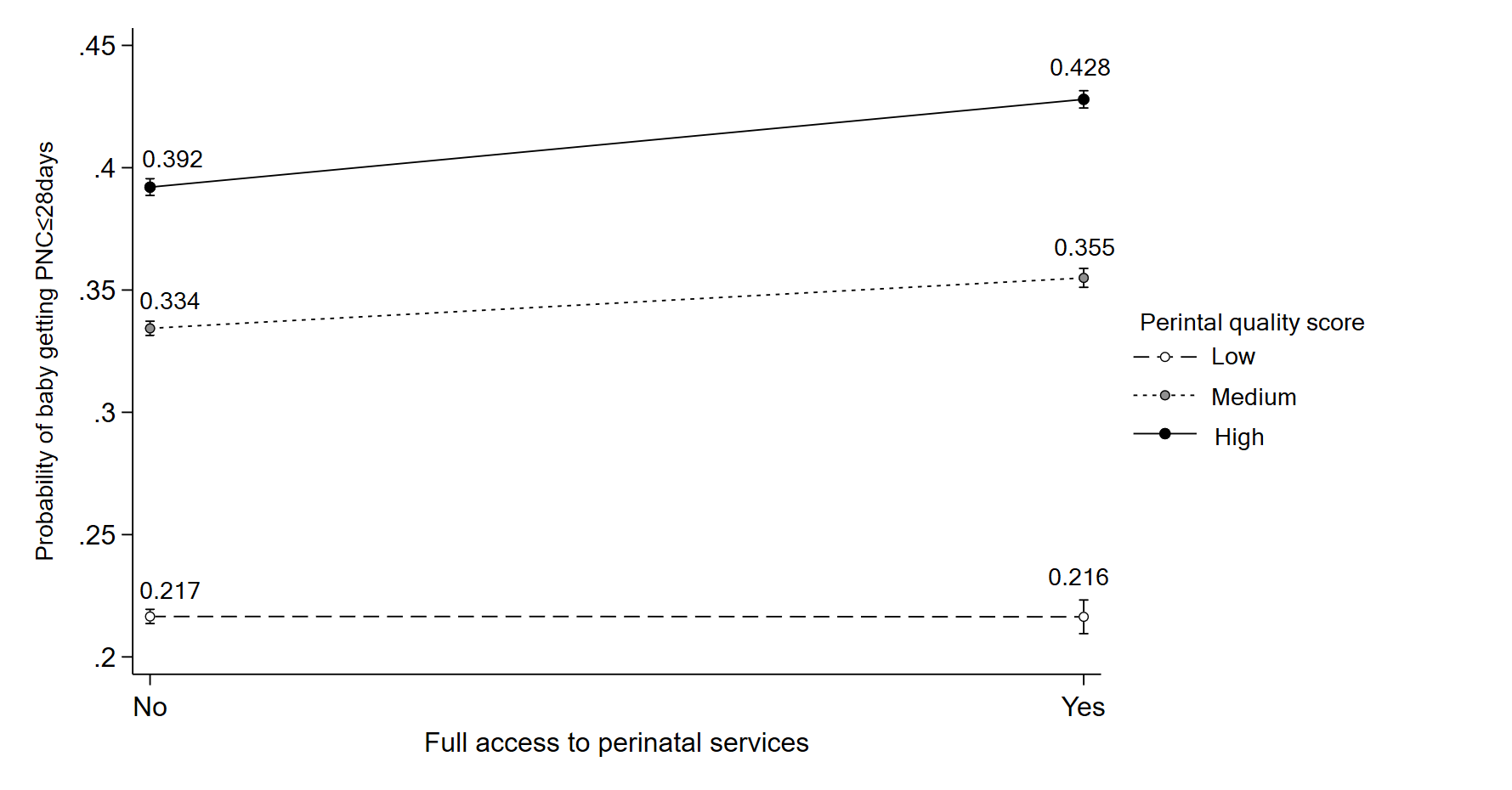


Figure A. Probability of babies receiving a postnatal check within 28 days when mother did and did not fully utilize perinatal services, across low (bottom 33.3%), medium (medium 33.3%) and high (top 33.3%) composite quality score group (post-estimated from Model 3).


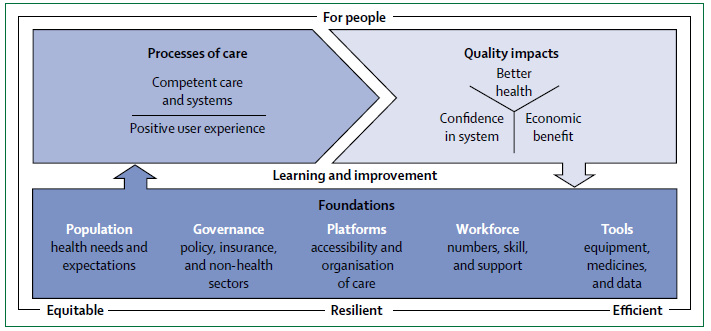


Figure B. High-quality health system framework suggested by Lancet Global Health Commission (2018)(Kruk, M.E., et al, 2018)
